# Revisiting Centrioles in Nematodes—Historic Findings and Current Topics

**DOI:** 10.3390/cells7080101

**Published:** 2018-08-08

**Authors:** Anna Schwarz, Prabhu Sankaralingam, Kevin F. O’Connell, Thomas Müller-Reichert

**Affiliations:** 1 Experimental Center, Faculty of Medicine Carl Gustav Carus, Technische Universität Dresden, 01307 Dresden, Germany; Anna.Schwarz1@tu-dresden.de; 2 Laboratory of Biochemistry, National Institute of Diabetes and Kidney Diseases, National Institutes of Health, Bethesda, MD 20892-0830, USA; prabhu.sankaralingam@nih.gov

**Keywords:** centrosome, centriole, *C. elegans*, Boveri

## Abstract

Theodor Boveri is considered as the “father” of centrosome biology. Boveri’s fundamental findings have laid the groundwork for decades of research on centrosomes. Here, we briefly review his early work on centrosomes and his first description of the centriole. Mainly focusing on centriole structure, duplication, and centriole assembly factors in *C. elegans*, we will highlight the role of this model in studying germ line centrosomes in nematodes. Last but not least, we will point to future directions of the *C. elegans* centrosome field.

## 1. Introduction

“*In der ruhenden Zelle besteht ausserhalb des Kerns in der Zellsubstanz ein specifisches Körperchen, das ich ››Centrosoma‹‹* […] *nenne*.”

[1]

“*In the resting cell outside of the nucleus there is a specific corpuscle, which I will term ››Centrosoma‹‹*.”

[1]

“*Gleichwohl enthält nun dieses Gebilde […] noch ein viel kleineres Körperchen, das von mir zuerst im Ascaris-Ei aufgefundene ››Centralkorn‹‹. Ich gebrauche für dieses Gebilde fortan neben dem Wort ››Centralkorn‹‹ den* […] *Terminus ››Centriol‹‹* […].”

[2]

*“There is also a much smaller little corpucle, the ››Centralkorn‹‹, which I discovered in* Ascaris *eggs. In parallel to the word ››Centralkorn‹‹, I will use the term ››Centriol‹‹* […].”

[2]

More than a hundred years ago, centrosomes and centrioles were discovered by Theodor Boveri, but fundamental questions pertaining to centrosome duplication and function remain to be answered. For instance, we still do not understand precisely how the centrosome is duplicated once and only once during the cell cycle. We have not deciphered the fine structure of the pericentriolar material (PCM) around the centrioles and how the centrioles function in organizing the PCM.

Boveri’s major discoveries on centrosomes were made using the early embryo of *Ascaris megalocephala* (now *Parascaris equorum*) for his light microscopy studies [3,4]. Today, nematodes are still favored by modern cell biologists, with the non-parasitic *Caenorhabditis elegans* emerging as a major genetic model system. This small free-living worm has contributed significantly to our knowledge of centrosome biology, as it offers a wide-range of tools for manipulating gene function and is highly amenable to various light and electron microscopy techniques. Undoubtedly, work in *C. elegans* will continue to contribute to a deeper understanding of the role of the centrosome in animal cell division and early development.

In this review, we will give a short summary on Boveri’s pioneering work on centrosomes, as he laid the scientific foundation for all future work on centrosome biology. We will then highlight some open questions that were posited by Boveri at the time and which are still under investigation in *C. elegans* and other species. We will also briefly review current work on centrosomes during embryonic mitosis in *C. elegans*. Last but not least we will outline future directions of the *C. elegans* centrosome field.

## 2. The Discovery of the Centrosome by Theodor Boveri—Early Observations

**Figure 1:**
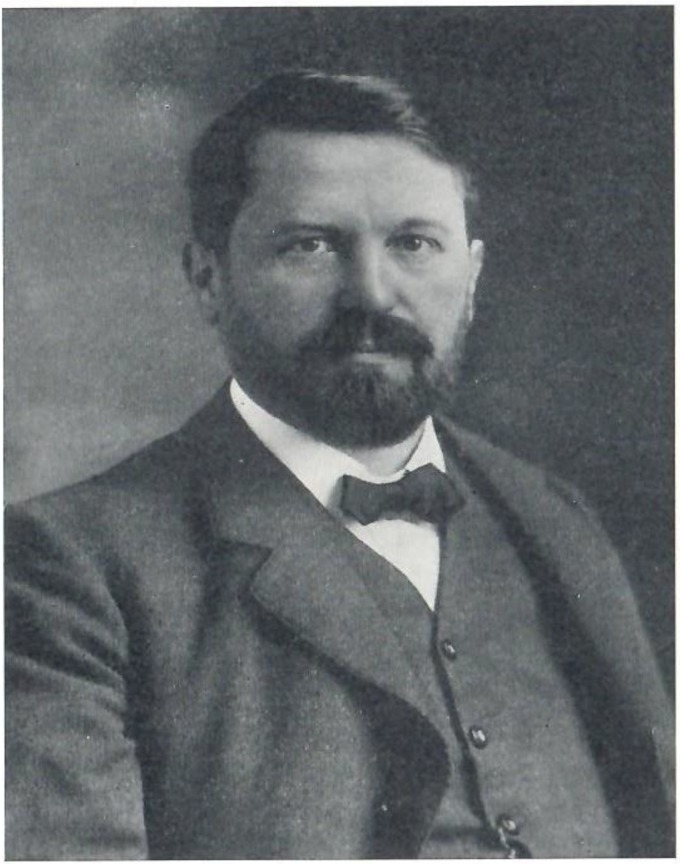
Theodor Boveri, the biologist and ‘father’ of centrosome research. Boveri was born in 1862 in Bamberg, Germany. After studying Anatomy and Biology he became one of the leading scientists of cell biology of his time. Reproduced from [3].

**Figure 2:**
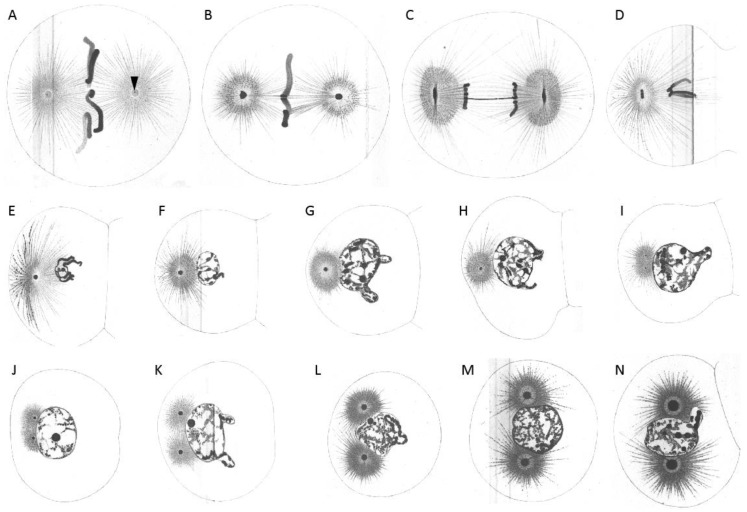
Boveri’s research on the first cleavage in the early embryo of *Ascaris megalocephala* to demonstrate the centrosome ’cycle’. Embryos were stained with iron haematoxylin. (**A**) Bipolar spindle with spherical centrosomes. Within the centrosomes, centrioles were drawn as small black dots (arrowhead). (**B**) Arrangement of chromosomes at the metaphase plate. (**C**) Separation of chromosomes and change in the shape of centrosomes from spherical to biconvex-discoidal. (**D**) Constriction between forming daughter cells, start of centrosome flattening. (**E**–**H**) Primary blastomeres. Centrosomes become spherical again (**E**), but smaller compared to the one-cell embryo. The nuclear vesicle is forming and the centrosome starts to divide again (**F**,**G**). (**I**) Separated centrosomes remain close to each other with the two asters clearly visible. (**J**,**K**) Daughter centrosomes move apart while growing in size. (**L**) The growing spherical centrosomes are completely separated. (**M**,**N**) Centrosomes move to opposite sides of the nucleus until they form a bipolar second spindle with full-sized centrosomes. The centrosomes show a ‘core’ and an outer layer. Reproduced from [2].

**Figure 3:**
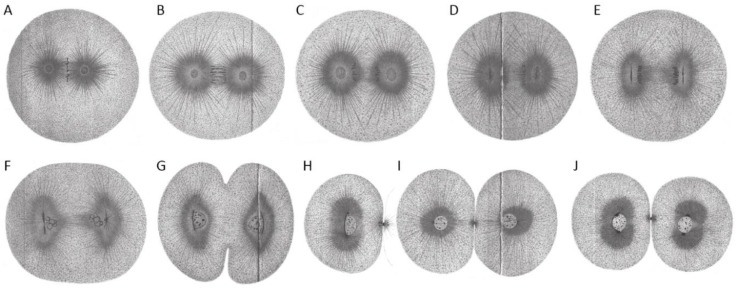
First cleavage in the early embryo of *Echinus microtuberculatus*. Sea urchin embryos were fixed in picric-acetic acid and stained with iron haematoxylin. (**A**) Chromosomes are arranged at the metaphase plate (equatorial plane). Centrosomes are spherical. (**B**) Centrosomes increase in size while homologs become separated. (**C**) Centrosomes start to flatten in the direction parallel to the division axis. (**D**) Flattened centrosomes change to discoidal form. (**E**) Centrosomes adopt a discoidal shape. (**F**) Spindle and egg elongate. Chromosomes are visible and centrosomes start to divide. (**G**) Cytokinesis of the embryo starts. (**H**,**I**) Embryonic daughter cells are formed (side-view (**H**) and cross-section/top-view (**I**) according to division axis). (**J**) Each daughter cell contains a nucleus and two centrosomes. Reproduced from [2].

Theodor Boveri (1862–1915), a German biologist working at the University of Würzburg Germany, introduced the term ‘centrosomes’ as early as 1887 (Figure 1). Boveri studied cell division in different organisms from the 1880s until the beginning of the 20th century. Through this work, he is recognized as the ‘father’ of centrosome research. He observed cell division in fertilized eggs of the nematode *Ascaris megalocephala* (Figure 2) and the sea urchin *Echinus microtuberculatus* (Figure 3). Early on, he recognized the extrusion of polar bodies after fertilization of the oocyte and the relevance of polar bodies for the subsequent mitotic cell division [5]. After a detailed analysis of the female meiotic spindles in fertilized oocytes of the earthworm *A. lumbricoides* and the nematode *A. megalocephala* [6], he observed the first mitotic spindle in an early *A. megalocephala* embryo and termed it ‘first cleavage spindle’ [7]. Here for the first time, he noticed two small refractive bodies at the poles of the mitotic spindle, which he called *polar corpuscle* (“Polkörperchen”) according to their position within the cell (not to be confused with the polar bodies that are extruded during female meiosis) [7]. At this time, he already described those “polar corpuscles” as the radiation centers of spindle filaments (known today as microtubules), which are surrounded by a bright halo [7]. The scientific term “centrosome” for this “polar corpuscle” was then introduced later in 1887 [1]. It should be noted here that the Belgian scientist van Beneden almost simultaneously described and published a *corpuscule central* [8] for same structure called *centrosome* by Theodor Boveri [4]. Here, however, we will focus entirely on the pioneering work of Theodor Boveri.

Boveri described the centrosome as a discrete cell organelle and dynamic center, which could divide, grow, and operate as an “attractive center” for the surrounding “Archoplasma”, which he defined as a spherical accumulation of a dense granular substance surrounding the “centrosome”. Furthermore, he postulated that the division of the centrosome initiates the following cell division. He also observed that the mature oocyte lacks centrosomes, while the fertilizing spermatozoon contains a single centrosome, which can divide after fertilization. From this he concluded that spermatozoa do not have any archoplasma [1]. Importantly, a centrosome-specific staining procedure was not available at the time. A popular method to stain centrosomes was the iron haematoxylin staining of Martin Heidenhain. Nevertheless, it was mentioned that this method does not just stain centrosomes but other components in the cell like chromatin [9] and protoplasmatic granules [10]. Boveri commented that the combination of centrosome-specific staining and expected cellular position would allow positive identification of the centrosome [2].

Several questions arose during his following studies with *E. microtuberculatus* [10]. For instance, it was not clear whether the centrosome belonged to the protoplasm of the cell or to the nucleus, and whether the centrosome was composed of nuclear or protoplasmic elements, or a mixture of both. Considering that the centrosome is an independent and permanent cell organelle comparable to the chromosomes, the question of how the centrosome evolved and took its shape was, of course, unanswered. In addition, it was still unknown how the divided centrosomes move apart, how the cell changes shape, and how the protoplasm splits.

After establishing that the centrosome is a dynamic and dividing cellular organelle, which mediates the division of the cell and the nucleus [6], Boveri declared in 1896 that the centrosome must communicate with the nucleus to form a connection between the chromosomes and the cell poles and that the nucleus is essential for the cell division [11]. As part of this idea, he also thought in detail about the initiation of cell division. He thought that the protoplasm takes an important role in the initiation of the division by changing its condition even if he did not understand the nature of this change. But he suggested that the protoplasm influences both the centrosome and the nucleus. Boveri was contemplating the contributions of the nucleus, the centrosomes, and the protoplasm and making predictions about their interdependencies. However, he excluded the scenario that the nucleus is responsible for the division and transformation of the centrosome [11].

After a time of dedication to the subject of the cellular and centrosomal division and their relation to each other, Boveri started to concentrate on the structure and mechanism of centrosome division. Assuming that a centrosome is duplicated by division into two centrosomes, he hypothesized that the mother centrosome is turned into two daughter centrosomes [2]. During duplication, he observed that the spherical centrosome assumes an ellipsoidal shape, divides and both daughter centrosomes grow from a flattened form into a spherical shape again before expanding.

In this context, Boveri observed an even smaller particle within the centrosome, which he first called “central granule” and later “centriole” [2]. Because this centriole splits as well, the cleavage into two centrioles was assumed as the first visible step of the centrosome division. Later he reported that the centrosomes can divide before their separation starts and that this stage of a doubled but not separated centrosome could be the centrosome ‘dauer’ stage of the cell [2].

The distinction between centrosome and centriole and the fact that it was hardly possible to distinguish both with the staining methods at the time, raised the question if the hitherto observed polar structures could be defined as the one or the other. Therefore, Boveri proposed that his stainings show centrosomes each with one centriole inside, even if the centrioles are not visible or optically distinguishable [2]. He defined the material the centriole is built of as “centroplasma” [2] and speculated on the relationship between the centrosome and the formation of the radial system of the surrounding sphere. He compared the centriole to a magnet, which attracts the surrounding protoplasm thus organizing it into a radial structure called the sphere. Of course, it was unclear how the centriole managed to attract protoplasm.

After the initial description of the bipolar spindle in *Ascaris megalocephala* [6,7], Boveri also thought about the establishment of bipolarity [2]. He proposed that the centrosome must split actively and suggested that both daughter centrosomes have to separate to form the two poles of the spindle before they induce the formation of the radiation around the poles [2]. Nevertheless, he concluded that centrosomes operate in a regulatory way rather than functioning as mechanical, structurally static organelles.

Today, there are still a number of fundamental open questions in the field. Indeed, we know that centrosomes are dynamic organelles, which vary in size, form, and structure during the cell cycle. We also know that centrosomes consist of ‘centroplasm’ (now called pericentriolar material, PCM) with a pair of centrioles at their center. However, we do not completely understand how the PCM is attracted and anchored to centrioles. While we have some insight into the mechanisms that ensure the production of one daughter per mother per round of duplication, we still do not completely comprehend how the cell achieves this level of accuracy. We also do not fully understand all of the molecular interactions that are necessary to specify the centriole’s final form, how centriole length is determined, and if the mother centriole might influence the structure of its daughter. These and other questions are being addressed by modern cell biologists, many of which, like Boveri have chosen to exploit the advantages of nematodes.

## 3. Return to Nematodes

In the early nineteen sixties, Sidney Brenner chose the non-parasitic soil nematode *C. elegans* as a model system to address questions related to development, cell division, and neurobiology. Like Boveri before them, modern cell biologists found the large unpigmented cells of the early nematode embryo an ideal specimen in which to study the mechanisms of cell division. Centrosomes, spindles and nuclei are all easily detectable using differential interference contrast microscopy. The first mitotic spindle is large (~14 μm in length at metaphase) as are the centrosomes, which nucleate massive arrays of astral microtubules. Importantly, throughout the early development of *C. elegans*, these visible cellular constituents exhibit a stereotypical pattern of movement, growth, and morphological changes, allowing geneticists to screen for mutants with specific cell division defects. Among the valuable cell division mutants identified in these screens are those with defects in the structure and behavior of germ-line centrioles.

**Figure 4:**
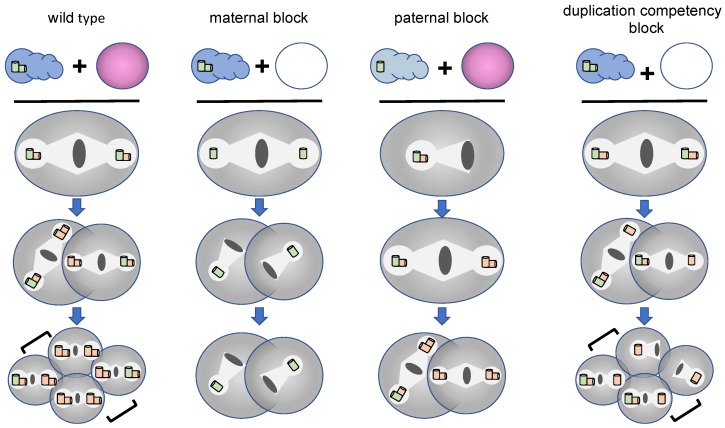
Patterns of spindle assembly in centriole duplication mutants. Spindle assembly phenotypes caused by different types of centriole assembly defects are depicted. The cellular composition of sperm (blue) and eggs (pink) that give rise to a particular spindle assembly pattern are shown at the top. Sperm centrioles are shown in green while centrioles generated in the embryo are shown in orange. Gametes drawn in lighter colors indicate the absence of a centriole assembly factor in either the maternal or paternal germ line. Brackets indicate sister cells in four-cell embryos. Wild-type embryos inherit a centriole pair from sperm that is duplicated faithfully during each cell cycle, giving rise to only bipolar spindles with centriole pairs at the poles. In a maternal block to duplication the sperm centriole pair is not duplicated in the embryo yielding a bipolar spindle with single centriole at each pole during the first cell cycle and monopolar spindles thereafter (bi/mono/mono pattern). In a paternal block, sperm donate a single centriole that is duplicated in the embryo yielding a monopolar spindle with a centriole pair at its pole during the first cell cycle, followed by bipolar spindles during subsequent cell cycles (mono/bi/bi pattern). In embryos exhibiting a maternal block to centriole maturation, the sperm centrioles duplicate normally but the daughters are unable to produce their own offspring. This results in the appearance of monopolar spindles during the third cell cycle (bi/bi/mixed mono pattern).

As it turns out, mutants with defects in the formation of germ-line centrioles possess some common properties that allowed them to be easily distinguished from other cell division mutants; this opened the way for rapid advances in understanding centriole duplication and other aspects of centrosome biology. First, all such mutants exhibit a maternal-effect embryonic lethal phenotype, owing to the fact that it is the mother that provides all of the factors necessary for the early embryonic divisions. That is, transcription is nearly absent in the early embryo, and thus essentially all transcripts and proteins present in the embryo are of maternal, and not zygotic, origin. Second, most of the germ-line centriole mutants exhibit a distinctive pattern of spindle assembly whereby the one cell embryo assembles a bipolar spindle, divides normally, and then assembles monopolar spindles at the two-cell stage (Figure 4) This bipolar/monopolar spindle assembly phenotype is diagnostic for a centriole duplication defect and arises because the zygote inherits its first pair of centrioles from the sperm. These sperm centrioles can separate and organize a bipolar spindle in the one cell embryo. However, in the absence of maternal factors essential for centriole duplication, each cell of the two-cell embryo inherits a single centriole and assembles a monopolar spindle.

The bipolar/monopolar pattern of spindle assembly that arises from a maternal centriole duplication defect is not the only phenotype conferred by a failure to properly duplicate germ line centrioles. It is also possible to create a paternal block in centriole duplication (Figure 4). In this case, centrioles fail to duplicate during the male meiotic divisions, giving rise to sperm that carry only a single centriole. If these sperm fertilize a wild-type egg, the resulting embryo will duplicate centrioles normally but during the first cell cycle will assemble a monopolar spindle organized around a centriole pair followed thereafter by bipolar spindles (monopolar/bipolar pattern of spindle assembly). Recently, another pattern of spindle assembly has been described that results not from a failure to assemble centrioles but from a failure of centrioles to attain duplication competency [12]. In this instance, when a wild-type sperm fertilizes an egg lacking the centriole maturation factor SAS-7, the two normal sperm centrioles duplicate to produce bipolar spindles during the first two cell cycles (Figure 4). However, daughter centrioles fail to mature, are unable to form their own daughters, and give rise to monopolar spindles during the third cell cycle (bipolar/bipolar/mixed monopolar pattern). Thus, the exact pattern of spindle formation can be highly informative, allowing researchers to gain important insights into the nature of the various centriole duplication defects.

## 4. The Structure of *C. elegans* Germ-Line Centrioles

Nearly all of what we know about the structure and duplication of centrioles in *C. elegans* derives from studies on the germ-line centrioles of the early embryo and sperm. These germ-line centrioles are structurally similar to those found in other species; they possess the signature of nine-fold symmetry and contain an inner scaffolding structure and an outer wall of microtubules. These germ-line centrioles, however, are smaller and simpler in structure than the centrioles of most metazoans. Compared to vertebrate centrioles, which are approximately 250 nm in diameter and 500 nm in length, *C. elegans* centrioles are just 100 nm in diameter and 150 nm in length [13,14]. Their outer wall is composed of nine singlet microtubules rather that the triplet or doublet microtubules observed in vertebrate cells and *Drosophila* embryos respectively [15].

The central scaffold also appears to be simplified in *C. elegans* centrioles. While most species possess an elegant cartwheel containing a central hub and nine radiating spokes, the worm counterpart appears as a simple central tube with a diameter of 70 nm and a length of 110 nm [14]. Despite these apparent differences in structure, the cartwheel and central tube are composed in large part by the same protein, SAS-6. SAS-6 has an N-terminal globular head with a fold reminiscent of the DNA repair protein XRCC4 [16,17], an extended C-terminal coiled-coil region, and an unstructured C-terminal tail. SAS-6 dimerizes through the coiled-coil region and dimers form higher order oligomers through head–head interactions. The cartwheel is thought to form through the stacking of flat disks, each composed of nine SAS-6 dimers arranged in a circle with the head regions forming the hub and the outward emanating coiled-coil regions forming the spokes [18].

While the structure and self-assembly properties of SAS-6 provide a satisfying model for the molecular architecture of the cartwheel, how SAS-6 might form a central tube was initially puzzling. However, Hilbert et al. found that *C. elegans* SAS-6 dimers could oligomerize in vitro into spiral structures, and molecular modeling indicated that ceSAS-6 could plausibly form a spiral with 4.5-fold symmetry, meaning that nine dimers could oligomerize to form two complete turns of the spiral [19]. Supporting this model, evidence of a hub-like structure within the central tube has recently emerged. Sugioka et al. noted a smaller inner tube in TEM micrographs of worm embryonic centrioles [12]. Indeed, reexamination of earlier ultrastructural data also reveals the presence of this inner tube [20,21]. The diameter of the inner tube (18 nm) is similar to that of the central hubs of other species (16–25 nm), and similar to the average diameter (15 ± 3 nm) of ceSAS-6 spirals. Furthermore, these micrographs appear to show spokes radiating outward from an inner tube. Thus, *C. elegans* early embryonic centrioles may be organized around a cartwheel-like scaffold as in other species.

**Figure 5:**
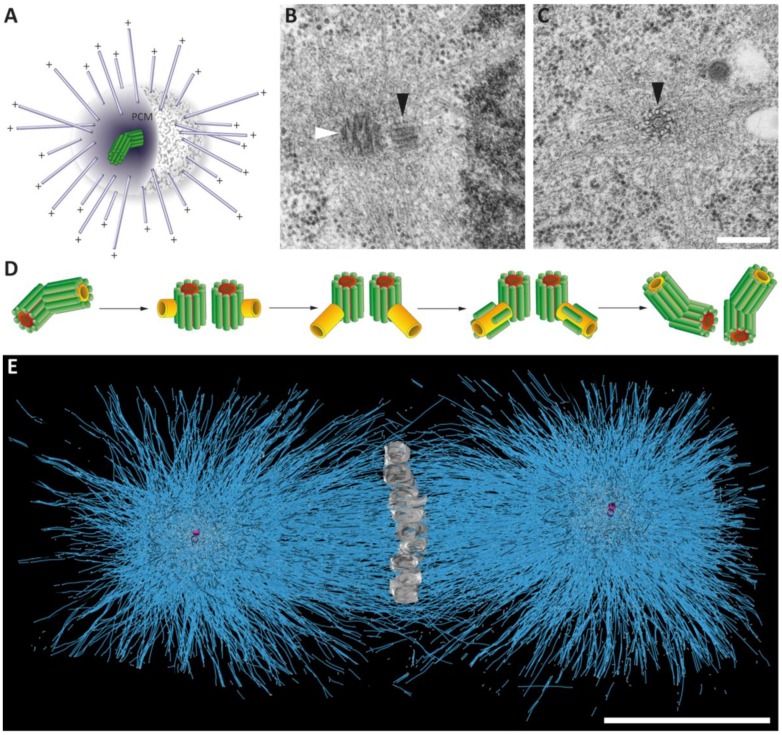
Centrosomes in the early *Caenorhabditis elegans* embryo. (**A**) Schematic representation of the centrosome with a pair of centrioles (green) within the pericentriolar material (PCM) and the nucleated microtubules (blue). The minus ends are anchored in the PCM, the plus ends (+) are growing out to cellular target sites. (**B**,**C**) Electron micrographs of *C. elegans* centrioles with surrounding PCM as observed in a 64-cell embryo. The mother (white arrowhead) and the daughter centriole (black arrowhead) are marked (**B**). *C. elegans* centriole in cross-section displaying a central tube (note the appearance of an inner tube within the central tube) and a peripheral arrangement of nine singlet microtubules (black arrowhead in **C**). Along the side of the microtubules, paddlewheel-like structures are twisted in a clockwise direction (black arrowhead). Modified from [31]. Scale bar, 250 nm. (**D**) Schematic drawing illustrating centriole duplication in *C. elegans*. Mother centrioles are shown with a red central tube, daughter centrioles with a yellow tube and centriolar microtubules in green. Centrosome duplication starts with a splitting of the centriole pair. New central tubes are formed perpendicular to the sperm-derived centrioles. The central tubes of the newly formed daughter centrioles elongate and new centriolar microtubules are assembled around the central tube. This results in two centrosomes each composed of one mother and one daughter centriole. Modified from [14]. (**E**) Full reconstruction of the first mitotic spindle in the one-cell *C. elegans* embryo. Chromosomes (grey) are arranged at the metaphase plate. The reconstruction shows the numerous microtubules (blue lines) of the first mitotic spindle. The centrioles are shown in magenta. Adapted from [32]. Scale bar, 5 µm.

As noted, the simplified germ-line centrioles of *C. elegans* also differ from the more complex centrioles of other species in that their outer wall is composed of singlet microtubules rather than doublets or triplets [22]. Perhaps not surprisingly, *C. elegans*, like *Drosophila*, lacks both delta-tubulin and epsilon-tubulin, which are required for the stable formation of triplet microtubules in human cells and various unicellular eukaryotes [23,24,25,26,27,28,29] (Figure 5). However, it should be noted that *Drosophila* germ-line centrioles possess triplet microtubules and thus these specialized tubulins are not absolutely required for triplet microtubule formation [15]. Not all *C. elegans* embryonic centrioles possess singlet microtubules however. In at least some sensory neurons that produce cilia, the centrioles possess doublet microtubules [30]. Presumably the elaboration of doublet microtubules in those centrioles destined to serve as basal bodies is a prerequisite for templating the doublet microtubules of the ciliary axoneme.

Appendages are another set of structural elements possessed by centrioles and include the distal and subdistal appendages that radiate outward from the centriole cylinder. Distal appendages promote membrane docking during ciliogenesis [33], while subdistal appendages anchor microtubule during interphase [34]. Such structures have neither been observed in *C. elegans* germ-line centrioles nor in those that function as basal bodies. Since worm centrioles only transiently function at the cell membrane to initiate ciliogenesis but are not required for completion of cilia assembly or maintenance [30], distal appendages might be dispensable in the worm. Similarly, the lack of an extended interphase during the early embryonic divisions might attenuate the need for subdistal appendages.

Recently, *C. elegans* germ-line centrioles have been found to possess a type of appendage termed a paddlewheel [12]. Unlike the distally-restricted appendages found in mammalian centrioles, these ~30 nm protrusions are found along the entire length of the centriole. Interestingly, when viewed from the distal end, the paddlewheels are twisted in a clockwise direction reminiscent of the clockwise twist of microtubule triplets in centrioles from other species. Thus *C. elegans* centrioles, like other centrioles, are inherently chiral structures. The role of paddlewheels has not been firmly established but their presence has been linked to the reproductive capacity of centrioles [12].

## 5. The Core Centriole Assembly Pathway

Pioneering work in *C. elegans* has led to the identification of a conserved set of core centriole assembly factors [35]. This set is presently comprised of SPD-2, ZYG-1, SAS-5, SAS-6, and SAS-4. In addition, the proteins SAS-7, PP2A, TBG-1 (γ-tubulin), and SPD-5 play important roles in centriole assembly in worm embryos [12,16,36,37]. The *zyg-1* (zygote defective) gene was the first of this set to be identified as essential for centriole assembly. ZYG-1 is considered to be the master regulator of centriole duplication [20] and the functional equivalent of vertebrate PLK4 (Polo-like kinase 4) and *Drosophila* Sak [38,39,40]. Sequence wise, ZYG-1 displays homology only in the N-terminal kinase domain. As a result, ZYG-1 was initially thought to lack the cryptic polo box (CPB) and polo box 3 (PB3) domains common to Plk4 family members. This led some to initially question whether ZYG-1 was evolutionarily related to Plk4 [41,42]. Jana et al. addressed this by using sensitive fold recognition programs to predict the presence of Plk4-like CPB and PB3 domains in the C terminus of ZYG-1 [43]. Ultimately, the crystal structure of the *C. elegans* CPB was solved, revealing an identical topology to that of the *Drosophila* and human Plk4 CBP [44,45]. Thus, ZYG-1 is a bona fide, yet divergent, member of the Plk4 family.

ZYG-1 is present at centrioles throughout the cell cycle [36] with its level peaking in anaphase [20]. ZYG-1 recruitment to the mother centriole is controlled by its centriole receptor SPD-2 (spindle defective) [14,46], a coiled-coil protein that localizes to both centrioles and PCM [47,48]. Accordingly, SPD-2 and its homologs also play a critical role in PCM formation [49,50,51,52]. A short acidic stretch of amino acids within the N-terminal region of SPD-2 interacts with basic cleft of the CPB domain of ZYG-1 through electrostatic interactions [44]. This mode of interaction is conserved across species, including humans [45,53] and *Drosophila* [44]. The human homolog of SPD-2 is known as Cep192 and was discovered in a proteomic analysis of interphase centrosomes [54]. In humans, Cep192 cooperates with another protein called Cep152 to recruit Plk4 [45,53,55,56]. Like Cep192, CEP152 possesses an N-terminal acidic region that interacts with the Plk4 CBP domain [45,53]. While worms lack a Cep152 ortholog and rely only on SPD-2 for Plk4 recruitment, in flies, SPD-2 is dispensable as the Cep152 ortholog Asterless serves as the sole centriole receptor for Plk4 [44]. Thus, while ZYG-1/Plk-4 is recruited in all three species through a similar type of molecular interaction, the identity of the receptor molecules themselves differs between species.

Once positioned at the site of centriole assembly, ZYG-1 then recruits the central scaffold components SAS-5 and SAS-6 (spindle assembly abnormal). As mentioned above, SAS-6 is a coiled-coil domain containing protein that was discovered in a genome-wide RNAi screen [36,57]. SAS-5 in contrast was identified in a forward genetic screen [58]. Like SAS-6, SAS-5 is also a coiled-coil domain containing protein. Ana2 [59,60] and STIL [61] are fly and human orthologs of SAS-5, respectively. Except for short stretches of alpha helical regions, SAS-5 is mostly disordered. Structural analysis of these domains revealed that they facilitate higher order oligomerization of SAS-5, necessary for centriole duplication [62,63].

SAS-5 and SAS-6 form a complex in the cytoplasm [57] that is recruited to the site of centriole assembly through a direct physical interaction between ZYG-1 and SAS-6 [64]. The kinase activity of ZYG-1 is dispensable for initial recruitment of the SAS-5/6 complex but it is needed for stable incorporation of SAS-6 into the nascent centriole. The integrity of the cytoplasmic SAS-5/6 complex is essential for centriole assembly as the two proteins are mutually dependent for their localization to procentrioles and neither protein can localize in the presence of mutations that disrupt complex formation [36,58,64]. Curiously, while SAS-6 is stably incorporated into the centriole, SAS-5 freely exchanges with its cytoplasmic pool [57,58]. Similar to *C. elegans* ZYG-1, human and *Drosophila* Plk4, are responsible for recruiting SAS-5 and SAS-6, however, some of the details of recruitment differ from that of worms. Specifically, in humans and flies, the kinase activity of Plk4 plays an important role in the recruitment of SAS-6; Plk4-dependent phosphorylation of specific residues within the STAN domain of STIL/Ana2 is necessary for it to interact with and recruit SAS-6 to nascent centrioles [65,66,67,68,69,70]. Thus, while a critical role for Plk4-mediated phosphorylation has been established in vertebrates and flies, the role of ZYG-1 mediated phosphorylation during centriole assembly in worms remains unresolved.

Incorporation of the SAS-5/6 complex leads to assembly of the central tube, which as discussed above imparts the nine-fold symmetry that is characteristic of centrioles. The nascent central tube is initially unstable [14] and only becomes stabilized later around the time that microtubule singlets are added to the outer wall. This terminal step of centriole assembly is dependent on SAS-4, another coiled-coil protein. In embryos depleted of SAS-4, the central tube can form and elongate to full length but appears to lose integrity shortly thereafter [14].

*C. elegans* SAS-4 is distantly related to human CPAP and *Drosophila* SAS-4 [71,72,73]. SAS-4-related proteins have a largely disordered N-terminus, a central coiled-coil domain and a structured C-terminal T complex protein 10 (TCP) domain, also known as a G (glycine rich)-box. SAS-4 localizes to the outer wall of the centriole and controls centriole size and pericentriolar material (PCM) accumulation [71,74,75]. The TCP domain of SAS-4 targets the protein to centrioles by binding SAS-5 [75,76,77]. SAS-4 constructs that lack the TCP domain localize diffusely to the PCM [77], suggesting that SAS-4 may play a role in organizing the PCM around centrioles. Indeed, it has been shown that the disordered N-terminus can bind microtubules and other PCM proteins [74,78,79,80], while the TCP domain interacts with SAS-5, thus tethering the PCM to centrioles [74,77,81].

In vertebrates, centriole assembly and the conversion of a daughter centriole to a mother are separable events [82]. Recent findings reveal the same is true in worms. Sugioka et al. recently found that the centriole-associated factor SAS-7 is required for centrioles to become replication competent [12]. Specifically, they found that in the absence of a cytoplasmic pool of SAS-7, wild-type centrioles can invariably produce daughter centrioles but the SAS-7-deficient daughters frequently fail to produce their own progeny. SAS-7 is required for the initiating event of centriole assembly, the recruitment of SPD-2 to the mother centriole, and achieves this through a direct physical association with SPD-2. Thus, conversion of a daughter to a mother centriole coincides with the ability to recruit SPD-2.

SAS-7 is a coiled-coil protein but does not have any significant sequence homology to proteins outside nematodes. Despite this, we noticed that its temporal localization pattern is reminiscent of CEP152 in humans [53] and Asl in *Drosophila* [83]. Just like CEP152 and Asl, SAS-7 is required for competence of daughter centrioles for next round of duplication.

## 6. Other Centriole Assembly Factors

In addition to the main players described above, other factors make important contributions to the centriole assembly process. Depletion of SPD-5, a major structural component of the PCM, interferes with centriole formation [36], suggesting that the PCM facilitates daughter centriole assembly. PCM could function by concentrating assembly factors within the vicinity of the assembling centriole [84]. Indeed, this same study found that depleting γ-tubulin, a PCM constituent involved in nucleating microtubules, produced an effect similar to that of depleting SPD-5 [36]. Although it has yet to be shown, γ-tubulin might participate in centriole assembly by nucleating the formation of the outer wall microtubules. In this context it is interesting to note that depletion of either SPD-5 or γ-tubulin causes an elongation of centriolar microtubules [85]. Thus, the PCM might produce a favorable micro-environment for proper centriole assembly by concentrating γ-tubulin and possibly other centriole assembly factors.

Lastly, a host of other factors contribute to the fidelity of centriole duplication by regulating expression of the main components of the pathway, thereby ensuring that each mother centriole produces one and only one daughter during each round of duplication. Among these are the phosphatases PP1 and PP2A. Loss of PP1 itself or either of the PP1 regulators I-2 or SDS-22 leads to the over expression of ZYG-1 and the production of multiple daughter centrioles [86]. Conversely, loss of PP2A activity leads to the under expression of ZYG-1 and SAS-5 and a failure in centriole duplication [37,87]. PP2A most likely functions by counteracting the activity of the SCF ubiquitin ligase complex which targets phosphorylated ZYG-1 [86], Plk4 [88,89,90,91,92,93], and STIL [94] for destruction.

Considering the entire body of knowledge, it is clear that multiple mechanisms contribute to the regulation of centriole assembly in *C. elegans* embryos. It is likely that additional regulatory inputs will be identified and that these pathways cooperate to ensure that centriole number is maintained from one generation to the next.

## 7. PCM Recruitment

While we now have a fairly good understanding of the ultrastructure and duplication of the *C. elegans* centriole, and the general roles of the various centriole assembly factors, much less is known about the role of the centriole in recruiting the PCM. In addition, we know very little about the composition and ultrastructure of the PCM itself. In electron micrographs of high-pressure frozen and freeze-substituted early embryos, centrioles are embedded in an electron-dense material of amorphous structure [13,32]. The mother centriole is always located within the center of the centrosome, while the daughter centriole is positioned more at the periphery [32]. Cryo-electron microscopy has been used rather recently to image networks of SPD-5 assemblies at high resolution [95]. This approach, however, did not reveal a filamentous organization of this major *C. elegans* PCM scaffold component. A clue as to the nature of the PCM comes from biophysical approaches explaining centrosomes as material occurring in two forms [96]: a dispersed soluble form in the cytosol and a concentrated form that appears to be generated through phase separation. In vitro data on the nature of the PCM was recently presented by reconstituting PCM-dependent microtubule nucleation [97]. Macromolecular crowding was reported to drive assembly of purified SPD-5 into spherical condensates in vitro. As observed in vivo, these condensates resembled the PCM morphologically and dynamically. Interestingly, the microtubule polymerase ZYG-9 (XMAP215 homolog) and the microtubule-stabilizing factor TPXL-1 (TPX2 homolog) could be selectively partitioned into the SPD-5 condensates. A high-resolution image of embryonic frozen-hydrated centrosomes, however, showing centrioles embedded in the PCM is still missing. One way to achieve this in vivo imaging might be the recently developed cryo-FIB-SEM approach for cryo-electron tomography [98]. This approach could potentially allow one to describe the ultrastructure of ‘native’ PCM.

## 8. Concluding Remarks

The past two decades have seen a rapid increase in our knowledge of centriole structure and assembly as well as PCM composition and behavior. *C. elegans* has been at the forefront of this advance and with its amenability to cytological and genetic approaches, we expect it will continue to serve as a preferred model for investigating centrosome biology. The development of new tools such as genome editing, conditional gene expression systems, and advanced imaging methods should allow *C. elegans* researchers to probe further into the details of centriole assembly and architecture, the mechanisms controlling centriole number and length, centriole-PCM interactions, as well as the in vivo structure and dynamics of the PCM. Such work will contribute to a deeper understanding of this important organelle, discovered more than hundred years ago in another nematode by Theodor Boveri.
